# VULNERABILITY, LIFE SATISFACTION, AND SOCIAL SUPPORT AMONG OLDER ADULTS: RESULTS FROM THE CLSA

**DOI:** 10.1093/geroni/igae098.0097

**Published:** 2024-12-31

**Authors:** Melanie Levasseur, Daniel Naud, Volker Cihlar, Frank Micheel, Andreas Mergenthaler, Lise Trottier

**Affiliations:** Université de Sherbrooke, Sherbrooke, Quebec, Canada; Université de Sherbrooke, Sherbrooke, Quebec, Canada; Federal Institute for Population Research, Wiesbaden, Hessen, Germany; Federal Institute for Population Research, Wiesbaden, Hessen, Germany; Federal Institute for Population Research, Wiesbaden, Hessen, Germany; Centre de recherche sur le vieillissement, CIUSSS Estrie - CHUS, Sherbrooke, Quebec, Canada

## Abstract

When experiencing, at a specific moment, multidimensional difficulties (physiological, psychological, socioeconomic or social) increasing the probability of being harmed or having coping challenges that negatively impact their lives, individuals in situations of vulnerability are at risk of reduced life satisfaction. Although not demonstrated yet, social support may mitigate this impact in older adults. This study aimed to examine the associations between situations of vulnerability, life satisfaction and social support, as well as the moderating effect of social support on the association between situations of vulnerability and life satisfaction among older women and men. Secondary analyses of the cross-sectional data on 21,491 respondents aged ≥65 from the Canadian Longitudinal Study on Aging were conducted, stratified by sex. Confirmatory factor analysis was used for identifying a latent vulnerability variable using physiological, psychological, socioeconomic and social indicators. Linear regression estimated the association between vulnerability and life satisfaction, and the moderating effect of social support. Respondents were aged between 65 and 89 (mean=73.4; standard deviation=5.8). For both sexes, reporting more depressive symptoms, chronic conditions and insufficient income best explained vulnerability, followed by dependence in basic activities of daily living, less social participation and living with fewer people. Vulnerability was associated with lower life satisfaction (p<0.001) for both sexes, and social support acted as a buffer against vulnerability (p<0.001) for women. Results confirm the multidimensionality of vulnerability. The buffering effect of social support in women reinforces recommendations concerning policies and interventions designed to increase opportunities of social connections, and further research is warranted for men.

